# The Angelina Jolie Effect in Jewish Law: Prophylactic Mastectomy and Oophorectomy in BRCA Carriers

**DOI:** 10.5041/RMMJ.10222

**Published:** 2015-10-26

**Authors:** Sharon Galper Grossman

**Affiliations:** Member of the Medical Advisory Board of The Lemonade Fund, Ra’annana, Israel; Consultant on the Medical Care of Oncology Patients, Ra’annana, Israel

**Keywords:** BRCA, prophylactic surgery, Jewish law

## Abstract

**Background:**

Following the announcement of actress Angelina Jolie’s prophylactic bilateral mastectomies and subsequent prophylactic oophorectomy, there has been a dramatic increase in interest in BRCA testing and prophylactic surgery.

**Objective:**

To review current medical literature on the benefits of prophylactic mastectomy and oophorectomy among BRCA-positive women and its permissibility under Jewish law.

**Results:**

Recent literature suggests that in BRCA-positive women who undergo prophylactic oophorectomy the risk of dying of breast cancer is reduced by 90%, the risk of dying of ovarian cancer is reduced by 95%, and the risk of dying of any cause is reduced by 77%. The risk of breast cancer is further reduced by prophylactic mastectomy. Prophylactic oophorectomy and prophylactic mastectomy pose several challenges within Jewish law that call into question the permissibility of surgery, including mutilation of a healthy organ, termination of fertility, self-wounding, and castration. A growing number of Jewish legal scholars have found grounds to permit prophylactic surgery among BRCA carriers, with some even obligating prophylactic mastectomy and oophorectomy.

**Conclusion:**

Current data suggest a significant reduction in mortality from prophylactic mastectomy and oophorectomy in BRCA carriers. While mutilation of healthy organs is intrinsically forbidden in Jewish law, the ability to preserve human life may contravene and even mandate prophylactic surgery.

## INTRODUCTION

In May 2013, the widely acclaimed Hollywood celebrity, Angelina Jolie, published an op-ed in *The New York Times* announcing that her mother, grandmother, and aunt had had cancer, that she had tested positive for the BRCA mutation, and that she had undergone bilateral prophylactic mastectomies to prevent breast cancer.^1^ This announcement led to what oncologists refer to as “The Angelina Jolie Effect,” a more than doubling in the demand for BRCA testing in women who would not otherwise have gone for testing, but were at high risk for carrying the mutation based on family history and therefore should have undergone genetic testing.^2^ In March 2015, Angelina Jolie published a second op-ed in *The New York Times* disclosing that she had undergone laparoscopic oophorectomy, removal of the ovaries to prevent ovarian cancer, and that she was receiving hormone replacement therapy to prevent the side-effects of premature menopause.^3^

## SCIENTIFIC BACKGROUND

Hereditary breast cancer accounts for 5%–10% of all breast cancer.^4^–^7^ The vast majority of inherited breast cancers are due to mutations in two breast cancer genes referred to as BRCA1 and 2.^6^ The risks of developing breast and ovarian cancer are higher in carriers of the BRCA1 mutation compared to carriers of BRCA2.^8^ In addition, cancer is more likely to occur at a younger age in carriers of BRCA1 mutations than in carriers of BRCA2. An average woman has a 12% lifetime risk of developing breast cancer.^9^ In a recent population-based study of Ashkenazi Jews in Israel, regardless of family history, the risks of developing breast cancer among BRCA1 and 2 carriers were 60% and 40%, respectively, and the risks of developing ovarian cancer were 53% and 62%, respectively.^10^ These results are consistent with the findings of a meta-analysis of 10 studies of patients in high-risk clinics which reported that the risks of developing breast cancer by the age of 70 in BRCA1 and 2 carriers are 57% and 49%, respectively, and the risks of ovarian cancer are 40% and 18%, respectively.^8^ These risks are significantly higher among women born more recently than among women born earlier, a birth cohort effect presumably due to modifications in non-genetic factors such as earlier menarche and later childbearing.^10^

Possible interventions for BRCA carriers might include increased surveillance, chemoprevention, and prophylactic surgery. Surveillance for breast cancer has consisted of MRI and mammogram beginning at age 25 or individualized to 10 years before the first cancer diagnosed in the family. While the addition of MRI to mammogram increases cancer detection rates and diagnoses cancer at an earlier stage, this strategy has not been shown to prolong survival in BRCA carriers.^11^–^14^ Surveillance for ovarian cancer has consisted of trans-vaginal ultrasound and blood tests for elevated tumor markers such as CA-125. However, this strategy has not been found to be effective.^15^,^16^ In fact, the National Cancer Institute does *not* recommend surveillance for ovarian cancer among BRCA carriers.

Another approach to reducing the risk of cancer among BRCA carriers is chemoprevention: tamoxifen to reduce the risk of breast cancer, and oral contraceptive pills to reduce the risks of ovarian cancer. Tamoxifen appears to be effective in reducing breast cancer in carriers of BRCA2 but not in carriers of BRCA1.^17^,^18^ The differential effect of tamoxifen may be due to the differential expression of the estrogen receptor in tumors of BRCA carriers. The BRCA1 carriers tend to develop estrogen receptor-negative breast cancers which do not depend on estrogen to grow, and the BRCA2 carriers tend to develop breast cancers that are estrogen receptor-positive and depend on estrogen to grow.^19^–^21^ Chemoprevention with tamoxifen has not been shown to prolong survival in BRCA2 carriers.^17^,^18^ Oral contraceptive pills have been shown to reduce the chances of developing ovarian cancer in BRCA carriers by 50% without increasing the risk of breast cancer, but this intervention has not been shown to reduce the chances of dying of ovarian cancer.^22^

## PROPHYLACTIC SURGERY

Prophylactic surgery consists of mastectomy, removal of the breasts to prevent breast cancer, and oophorectomy, removal of the ovaries to prevent ovarian cancer. Prophylactic mastectomy reduces the chances of developing breast cancer by 90% or more.^23^–^30^ In one series, there were no cases of breast cancer 3 years after prophylactic surgery.^29^ Prophylactic oophorectomy reduces the chances of developing ovarian cancer by 80% and can also reduce the chances of developing breast cancer by 50%.^31^–^35^ Carriers of the BRCA mutation may opt for mastectomy alone with surveillance of the ovaries (as Angelina Jolie chose to do between 2013 and 2015), prophylactic oophorectomy alone which will reduce the chances of developing both breast and ovarian cancer, or prophylactic mastectomy and oophorectomy (which Angelina Jolie ultimately chose to do). The ideal age to perform prophylactic oophorectomy is not known, but the recommendation is to complete childbearing by age 35–40 and then undergo prophylactic surgery as there is concern that delaying oophorectomy would increase the chances of developing ovarian cancer.^35^ In addition, the magnitude of the protective effects of oophorectomy in reducing the chances of breast cancer is greater the younger the age of oophorectomy.^29^,^36^ Oophorectomy at this age does cause premature menopause; however, as Angelina Jolie has illustrated, these symptoms can safely be managed with short-term hormone replacement therapy.^37^–^39^

How do BRCA carriers cope with prophylactic surgery? Emerging data would suggest that overall quality of life for BRCA carriers who undergo prophylactic surgery is not compromised by surgery. The BRCA carriers who undergo prophylactic surgery report high satisfaction with surgery and less cancer worry than BRCA carriers who opt for surveillance.^40^,^41^ However, prophylactic surgery may be associated with sexual dysfunction and menopausal symptoms which can be addressed by proper medical intervention. Although it was initially thought, based on theoretical modeling, that BRCA carriers who underwent prophylactic surgery would live longer than those who opted for surveillance, this was not based on actual prospective patient data.^42^ Recently, a number of studies have confirmed that BRCA carriers who undergo prophylactic surgery live longer than those who undergo surveillance.^29^,^35^,^43^ In one series, the chances of dying of ovarian cancer were reduced by 95% among BRCA carriers who opted for prophylactic oophorectomy compared to carriers who opted for surveillance, the chances of dying of breast cancer were reduced by 90%, and the chances of dying of any cause were reduced by 70%.^29^ Previously, it was assumed that prophylactic oophorectomy would reduce the chances of a BRCA carrier dying of ovarian cancer but that inducing premature menopause would be harmful and negate any beneficial effects of preventing ovarian cancer. Physicians believed that the BRCA carrier who opted for prophylactic oophorectomy was “trading” ovarian cancer for a host of new medical problems associated with entering premature menopause for no “net” benefit. Yet, the most recent studies show that, overall, BRCA carriers who undergo prophylactic surgery live longer than those who opt for surveillance, demonstrating that, regardless of what medical problems premature menopause may cause, the net effect of prophylactic surgery is that carriers who undergo such surgery live longer. The medical benefits clearly outweigh the risks.

## HALACHIC ISSUES

From a Halachic perspective, there are several concerns arguing against prophylactic surgery (Table 1). First of all, prophylactic surgery involves mutilation of a healthy organ. Secondly, removing a healthy organ—especially one that defines a woman’s sexuality and her appearance—may cause significant psychological distress, although the available quality of life data would suggest that from a psychological perspective this surgery is well tolerated.^40^,^41^

**Table 1:** Halachic Debate over Prophylactic Surgery.

Against	For
Mutilates healthy organ Causes psychological distress Inflicts a wound Destroys ability to be fruitful Violates prohibition against castration	Prolongs life

In addition, there are two other potential Halachic concerns regarding removal of ovaries. First, removal of ovaries may prevent the BRCA carrier from fulfilling the mitzvah to “be fruitful and multiply” (*pru u’revu*). Second, removal of ovaries may violate the prohibition against castration (*sirus*). Arguing in favor of prophylactic surgery are new, emerging, compelling medical data showing that BRCA carriers who undergo prophylactic surgery are less likely to die of cancer and more likely to live longer than women who opt for surveillance. Angelina Jolie’s decision prophylactically to remove her healthy breasts and most recently her healthy ovaries raises several Halachic questions including the following: (1) is there a Halachic obligation to prevent disease; (2) is it permitted for a BRCA carrier to remove a healthy organ; (3) does prophylactic surgery violate the prohibition against harming one’s body (*chovel)*; (4) does prophylactic oophorectomy prevent the BRCA carrier from fulfilling the mitzvah to procreate; (5) does prophylactic oophorectomy violate the prohibition against castration; (6) is it permitted to perform prophylactic surgery in a BRCA carrier; (7) is a BRCA carrier *obligated* by Halacha to undergo prophylactic surgery; and, lastly, (8) are we as a Jewish society, particularly in Israel, obligated to pay for prophylactic surgery in BRCA carriers?

### 1. Is There a Halachic Obligation to Prevent Disease?

In brief, the Halachic obligation to prevent disease can be based on several texts in the Torah. Rambam in *Hilchot Deot* cites a verse from Deuteronomy, “Only be careful, and watch yourselves closely,”^44^ as the basis for the Halachic obligation to prevent disease.^45^ Rambam explains that the Halachic basis for preventing disease is to give us the necessary tools to worship Hashem. A healthy body is a necessary condition for performing the commandments (*mitzvot*) and serving G-d. In addition, Rambam lists a series of medical interventions that we are obligated to adhere to in order to prevent disease, including eating only when hungry, drinking only when thirsty, going to the bathroom when necessary. This is not an exclusive list but a broad list with general applicability and fluidity. Presumably, as medical knowledge evolves, other medical interventions that are determined to prevent disease would fall under the list of interventions that we are obligated to perform to prevent disease and give us the strength to worship Hashem. Although Rav Moshe Feinstein interprets Rambam as a guideline rather than a mandate,^46^ Rabbi Bleich suggests that prophylactic surgery in BRCA carriers which clearly has been shown to prolong survival would fall into the list of medical interventions Halachically required to prevent disease.^47^

### 2. Is It Permitted for a BRCA Carrier to Remove a Healthy Organ for a Disease That She Does Not Have and May Never Develop?

Approximately 50% of BRCA carriers will develop breast cancer, and 50% ovarian cancer. Is it permitted to remove a healthy breast if 40% of women undergoing this surgery will never develop breast cancer? Is it permitted to remove healthy ovaries if 50% of carriers undergoing surgery will never develop ovarian cancer? Rav Feinstein addressed these questions when he was asked whether a woman undergoing hysterectomy could have her healthy ovaries removed at the time of surgery to prevent cancer of the ovaries in the future. It was estimated that the risk of developing ovarian cancer in the woman in question was 5%.^48^

Rav Feinstein concludes that it is permitted to remove the healthy ovaries of a woman who has a 5% risk of ovarian cancer in the future and that such surgery is for her benefit (*l’tovata*). If Rav Feinstein permitted the removal of the healthy ovaries of a woman with a 5% chance of developing ovarian cancer in the future, then it certainly should be permitted to remove the healthy ovaries of a BRCA carrier with a 50% chance of developing ovarian cancer in the future and the healthy breasts of a BRCA carrier with a 50%–60% chance of developing breast cancer in the future. Such surgery would also be for her benefit.

In this responsa, written in 1982, Rav Moshe expressed concern regarding the risks of complications from surgery and the psychological impact, on the woman in question, of removing healthy organs. Since that time, however, operative techniques have improved and complications have decreased. In fact, Angelina Jolie’s surgery was performed laparoscopically with minimal blood loss. In addition, recent data show that BRCA carriers who undergo prophylactic surgery tolerate surgery remarkably well from a psychological perspective.^40^,^41^ In light of the dramatic reduction in surgical complications which has occurred in the last 20 years and current data that prophylactic surgery does not cause significant mental distress, it would seem likely that if asked the same question today regarding the permissibility of removing healthy ovaries in a woman who carries a 50% risk of developing ovarian cancer, Rav Feinstein would be even more inclined to permit such surgery.

### 3. Does Prophylactic Surgery Violate the Prohibition against Harming One’s Body?

The prohibition against inflicting a wound or harming one’s body (*chovel)* comes from Leviticus, “Do not cut your bodies for the dead or put tattoo marks on yourselves.”^49^ When asked if a young girl could undergo cosmetic surgery, Rav Feinstein concluded that such surgery did not violate the prohibition against inflicting a wound since the intention of surgery was not to humiliate the patient but rather to make her more attractive.^50^ In other words, if the purpose of inflicting a wound is not to harm the patient or cause shame or humiliation (*biyzayon*), surgery would be permitted and for the benefit of the woman. Thus, extrapolating from Rav Feinstein’s responsa to the case of the BRCA carrier, the prohibition against wounding oneself would not apply to prophylactic surgery as the purpose of such surgery is not to humiliate the carrier, but to prolong her life. Prophylactic surgery would be for the benefit of the carrier (*l’tovata*). Interestingly, in the conclusion of his responsa, Rav Feinstein quotes the Talmud in *Ketubot* that states, “*Ein Isha eila leyofi*,”^51^ “a woman is only for beauty” and concludes that if the wound is inflicted to make the woman more beautiful the prohibition against wounding oneself is annulled. If, in fact, wounding oneself is abrogated for surgery that improves a woman’s appearance, perhaps Rav Feinstein would conclude that in a BRCA carrier undergoing prophylactic surgery, which deforms the appearance of the woman, the prohibition against wounding oneself persists. However, given that prophylactic surgery prolongs life, ultimately saving a human life (*pikuach nefesh*) would override the prohibition against inflicting a wound.

### 4. Does Prophylactic Oophorectomy Prevent the BRCA Carrier From Fulfilling Her Obligation to Procreate (*pru u’revu*)?

There is much Halachic debate regarding whether a woman has an obligation to procreate, and this is presented in great detail in Tractate *Kiddushin*. Rav Feinstein addresses this issue in his responsa regarding the woman who is to undergo removal of healthy ovaries at the time of hysterectomy.^48^ He explains that, even if the woman has an obligation to procreate, the woman in question had completed childbearing and removal of her healthy ovaries could not prevent her from fulfilling the obligation to procreate, assuming such an obligation exists. Similarly, the recommendation for BRCA carriers is to complete childbearing by the age of 35–40 years and then undergo prophylactic oophorectomy. Prophylactic oophorectomy in a BRCA carrier who has completed childbearing would not interfere with a woman’s obligation to procreate assuming such an obligation exists for her, as she has already fulfilled her obligation. Similarly, Angelina Jolie had completed childbearing when she underwent prophylactic oophorectomy. Prophylactic oophorectomy in a BRCA carrier who has not completed childbearing by age 40 may interfere with a woman’s obligation to procreate if such an obligation exists. However, as women age, they become infertile and, at some point, the BRCA carrier who has not completed childbearing will no longer be able to bear children. Prophylactic oophorectomy in a BRCA carrier who has not borne children but has become infertile would no longer interfere with a woman’s obligation to procreate assuming a woman is in fact obligated to procreate, because she is already infertile (*akara)*.

### 5. Does Prophylactic Oophorectomy Violate the Prohibition Against Castration?

The prohibition against castration is derived from Leviticus, “That which is bruised or crushed, torn or cut do not bring before Hashem…”^52^

There is a great deal of debate among Rabbinic scholars regarding the nature of this prohibition. Does it apply just to men or also to women? Is it Rabbinic or Biblical? Does it refer to surgical or medical castration? Rav Feinstein addresses these issues in his responsa regarding removal of healthy ovaries.^48^

Even if the prohibition against castration is Biblical and applies to women, he explains that the woman undergoing oophorectomy had already gone through menopause and had already naturally entered a medical/hormonal state of castration. Since the woman had already undergone menopause, removing her ovaries would not violate the prohibition against castration. She had already been autocastrated. It is not possible to castrate a eunuch, because a eunuch is already castrated. Thus, prophylactic oophorectomy in a post-menopausal BRCA carrier would not violate the prohibition against castration, since the ovaries of the BRCA carrier had already ceased to function and she had reached a hormonal state of castration prior to oophorectomy.

However, from a medical perspective, prophylactic oophorectomy in a BRCA carrier should ideally be performed before menopause as the risk of developing ovarian cancer increases with age and the protective effects of prophylactic oophorectomy in reducing the risk of breast cancer are only seen when such surgery is performed before the age of 50.^29^,^36^ The younger the BRCA carrier is when prophylactic oophorectomy is performed, the greater the reduction in risk of breast cancer.^29^,^36^ Hence, does prophylactic oophorectomy in premenopausal BRCA carriers, whose ovaries are still functioning, violate the prohibition against castration? Although the Genius from Vilnius (Vilna Gaon) felt castration in a woman, like a man, is forbidden by the Torah,^53^ Bach and Rav Shlomo Luria, in Yam Shel Shlomo, permit castration in a woman who cannot bear the pains of childbirth and there are no other alternative forms of contraception available.^54^,^55^ If the prohibition of castration is overridden by the pains of childbirth, it certainly should be abrogated if surgery would prolong life. A premenopausal BRCA carrier who undergoes prophylactic oophorectomy does not violate the prohibition against castration because the saving of human life ultimately trumps this and any other prohibition. In general, the saving of human life almost always overrides the commandments when the situation is immediately at hand (*le’faneinu*). The potential saving of human life, which does not exist at the time of decision-making, cannot override prohibitions. Rabbi Avraham Steinberg as well as Rabbi Zalman Nechemiah Goldberg and Rabbi Asher Weiss share this view that for a BRCA carrier the prohibition of castration is annulled by the saving of human life, because the BRCA carrier is in a condition of possible danger to life from conception when she inherited the BRCA mutation.

Thus, the Rabbis cited in this article believe that for a BRCA carrier the risk to human life is imminent and the prohibition against castration, or any other prohibition for that matter, may be annulled to save a human life (personal communication from Rabbi A. Steinberg to the author, May 2015).

### 6. Is It Permitted to Perform Prophylactic Surgery in a BRCA Carrier?

In 2006, Rabbi Moshe Tendler argued that such surgery was not permitted by Halacha. He said: “Although there is a Jewish legal imperative to protect life, it intersects here with the injunction against harming one’s own body. No one can fault a woman for running scared and doing these surgeries, but it isn’t a 100 percent guarantee” that she will not get these cancers.^56^

Rabbi Tendler, at that time, recommended against prophylactic surgery, preferring increased non-invasive surveillance.^57^ Given the most recent data showing that prophylactic surgery prolongs life and that surveillance does not (certainly surveillance for ovarian cancer has not been shown to be at all effective), the author contacted Rabbi Tendler to ask if his views have changed. Rabbi Tendler replied that based on the current data “the surgical approach would be the Halachic advisory^8^ and prophylactic surgery would be Halachically permitted” (personal communication from Rabbi M.D. Tendler to the author, May 2015). Rav Avraham Steinberg as well as Rav Zalman Nechemiah Goldberg and Rav Asher Weiss also believe that prophylactic surgery is permitted in a BRCA carrier (personal communication from Rabbi A. Steinberg to the author, May 2015).

### 7. Is a BRCA Carrier Obligated by Halacha to Undergo Prophylactic Surgery?

At a 2008 Yeshiva University medical ethics conference titled “Catching Cancer Before It Catches You” Rabbi Mordechai Willig concluded that a BRCA carrier would be obligated to undergo prophylactic surgery. He stated that surgeries should be done, may be “done and maybe from the Halachic perspective MUST be done in order to save lives.”^57^ Rabbi Yair Hoffman has argued that *kallah* teachers should be instructing all women to be tested at age 25 and for women who are BRCA-positive prophylactic surgery is Halachically recommended at age 35 for BRCA1-positive carriers and after 40 for BRCA2-positive women.^58^ Rabbi J.D. Bleich has said: “Any medically indicated prophylactic or diagnostic procedure fits with obligation posited by Rambam. Genetic testing, including BRCA should be regarded as Halachically mandated when results are likely to affect treatment and enhance longevity or well-being. One at risk is obligated to pursue all measures to ward off the disease.”^47^

### 8. Are We Obligated as a Jewish Society to Pay for Prophylactic Surgery in BRCA Carriers?

Rav Eliezer Waldenburg was asked about performing vision testing in children to prevent ophthalmologic disease.^59^ He explained that communal funds should be used to finance prophylactic examination based on “Love your neighbor as yourself.”^60^ Extrapolating to the case of BRCA carriers, if BRCA carriers are Halachically obligated to undergo prophylactic surgery, then we as a Jewish society would be obligated to fund both BRCA testing and prophylactic surgery in those who test positive.

## CONCLUSION

In summary, there is a clear Halachic obligation to prevent disease. It is permitted to remove a healthy body part to prevent disease in the future. Prophylactic oophorectomy interferes with obligations to procreate and prohibitions of castration. Oophorectomy after completing childbearing helps eliminate issues relating to “procreation.” When performed after menopause, prophylactic oophorectomy may obviate the prohibition against castration. Ultimately, saving a life overrides castration and any prohibitions including the prohibition against harming one’s body. Given the emerging data favoring prophylactic surgery, a growing number of Jewish arbiters believe that prophylactic surgery is Halachically permitted, with some positing that a BRCA carrier is obligated to undergo prophylactic surgery.

### Angelina Jolie’s Status in Judaism

Angelina Jolie’s very public medical journey has increased awareness of the BRCA mutation and the demand for testing in high-risk women who would not otherwise have been tested. In addition, she has increased interest in potentially life-saving prophylactic surgery. It is not possible to measure how many lives she has saved by making her very personal, medical odyssey public. Regardless of her other behaviors and politics, her decision to publicize her status as a BRCA carrier and her decisions to undergo prophylactic surgery make her worthy of the description *in Sanhedrin*, “Whoever saves one Jewish life is considered to have created an entire world.”^63^

Angelina Jolie has created many worlds, and for this we as a Jewish people must be eternally grateful.

## Glossary

G-d: Hebrew manner of writing the name of the Almighty in English
Hashem: G-d
kallah teachers: special instructors of Jewish women on Halachic requirements in marital life
Torah: first five books of Moses
rav: rabbi.
